# Description and complete genome sequence of *Mycobacterium venezuelense* sp. nov., a rapidly growing species recovered from a soft tissue infection

**DOI:** 10.3389/fmicb.2026.1780266

**Published:** 2026-04-02

**Authors:** Yrneh Yadamis Prado Palacios, Leonardo Bruno Paz Ferreira Barreto, Maria Carolina Sisco, Emilyn Costa Conceição, Abhinav Sharma, Francisco das Chagas de Sousa, Guilherme da Silva Lourenço Carvalho, Daisy Anne Barbosa de Lira, Bruna Moreira de Sousa Melo, Ricardo Pérez-Alfonzo, Javier E. Ortega Diaz, Franklin E. Claro Almea, Douglas Silva, Edson Machado, Sidra E. G. Vasconcellos, Philip Suffys, Maria Cristina da Silva Lourenço, Jacobus H. de Waard

**Affiliations:** 1 Departamento de Tuberculosis y Micobacteriosis, Instituto de Biomedicina Dr. Jacinto Convit, Universidad Central de Venezuela, Caracas, Venezuela; 2 Laboratório de Bacteriologia e Bioensaios em Tuberculose e outras Micobactérias (LBBTM), Instituto Nacional de Infectologia Evandro Chagas, Instituto Oswaldo Cruz, Rio de Janeiro, Brazil; 3 Laboratório Nacional de Computação Científica, Ministério da Ciência, Tecnologia, Inovação e Comunicações, Petrópolis, Brazil; 4 SAMRC Centre for Tuberculosis Research, Division of Molecular Biology and Human Genetics, Faculty of Medicine and Health Sciences, Stellenbosch University, Cape Town, South Africa; 5 Centro Clínico de Dermatología y Enfermedades Tropicales, Instituto de Biomedicina Dr. Jacinto Convit, Universidad Central de Venezuela, Caracas, Venezuela; 6 Laboratório de Biologia Molecular aplicada a Micobactérias, Instituto Oswaldo Cruz, Rio de Janeiro, Brazil; 7 Facultad de Medicina, Universidad Espíritu Santo, Samborondón, Ecuador

**Keywords:** drug susceptibility testing, MALDI-TOF, nontuberculous mycobacteria, partial sequencing, PRA-hsp65, WGS

## Abstract

We describe a rapidly growing mycobacterial (RGM) species closely related to *Mycobacterium wolinskyi*. The bacterium was isolated from three biopsies obtained from a 78-year-old Venezuelan woman who presented with a chronic superficial erythematous plaque in the right nasal cavity. The three isolates, LTG2003(1–3), exhibited identical phenotypic, biochemical, and molecular characteristics and showed susceptibility to amikacin and linezolid, intermediate susceptibility to cefoxitin and imipenem, and resistance to ciprofloxacin, clarithromycin, sulfamethoxazole-trimethoprim, and tobramycin. Conventional diagnostic methods, including biochemical assays, *hsp65* PCR restriction enzyme analysis (PRA-*hsp65*), the Genotype Mycobacterium CM v2.0 linear probe assay, and Sanger sequencing of the *16S rRNA*, *hsp65*, and *rpoB* genes, failed to identify the microorganism to the species level. MALDI-TOF mass spectrometry yielded genus-level scores suggestive of *M. wolinskyi*. Whole-genome sequencing confirmed that the isolates represent a new species. The type strain, designated LTG 2003-1 (WFCC 1312 = P7483 = CDBB-500-3), has been deposited in two internationally recognized microbial culture collections in Mexico and Brazil. The genome of this organism has also been deposited in GenBank as *Mycobacterium* sp. LTG2003 with accession number JBONDW000000000. Based on our findings, we propose that the LTG2003(1-3) isolates represent a new species, for which we propose the name *Mycobacterium venezuelense* sp. *nov*.

## Introduction

Nontuberculous mycobacteria (NTM) are environmental microorganisms that are generally nonpathogenic to humans, except in individuals with underlying immunosuppression or other predisposing conditions (Cook, 2010; Ratnatunga et al., 2020). Once considered of limited clinical relevance, NTMs are now recognized as important opportunistic pathogens, driven in part by increasing global detection rates and advances in diagnostic methods (Moore et al., 2010; Johnson and Odell, 2014; Donohue, 2018). Although pulmonary disease is the most common clinical presentation, NTMs are also responsible for infections of the skin, soft tissues, and postsurgical wounds, including cases reported in Venezuela (Weitzul et al., 2000; Piquero et al., 2004; Henkle and Winthrop, 2015; Torres-Coy et al., 2016; Zimmermann et al., 2017; Pérez-Alfonzo et al., 2020; Da Mata-Jardín et al., 2020; García-Ruza et al., 2024).

Rapidly growing mycobacteria (RGM), a subgroup of NTMs, were historically regarded as having low virulence (Falkinham, 1996; De Groote and Huitt, 2006; Mohananey et al., 2018). However, their clinical significance has increased, particularly among immunocompromised patients. RGMs are frequently associated with postsurgical and device-related infections, outbreaks in healthcare settings, and they often exhibit intrinsic or acquired multidrug resistance, complicating therapeutic management (van Ingen et al., 2012). Clinically relevant species include *Mycobacterium abscessus*, *Mycobacterium fortuitum*, and *Mycobacterium chelonae*, among others (Brown-Elliott and Wallace, 2002; Brown-Elliott and Philley, 2017).

Isolation and identification of RGMs remain challenging. These organisms may grow more slowly than other bacteria in polymicrobial samples and can therefore be missed or overgrown in routine cultures (Han et al., 2007). In addition, accurate species-level identification is difficult because traditional biochemical methods lack sufficient discriminatory power. Molecular approaches, including PCR–restriction enzyme analysis of *hsp65* (PRA-*hsp65*) and partial Sanger sequencing of the *16S rRNA*, *hsp65*, and *rpoB* genes, have improved diagnostic accuracy but remain insufficient to reliably differentiate rare, emerging, or closely related species. Although MALDI-TOF mass spectrometry (MS) has substantially increased the speed of mycobacterial identification, its performance is limited by the incompleteness of available reference spectral libraries, hindering the recognition of novel taxa (Wang et al., 2025).

The taxonomic classification of the genus *Mycobacterium* has undergone major revisions in recent years, including a proposal to divide the genus into five separate genera—*Mycobacterium*, *Mycolicibacterium*, *Mycolicibacillus*, *Mycolicibacter*, and *Mycobacteroides*—based on shared molecular markers (Gupta et al., 2018). Subsequent phylogenomic analyses, however, recommended retaining a single unified genus due to the clinical, diagnostic, and practical implications of renaming medically relevant species (Meehan et al., 2021). As both nomenclatures continue to appear in the literature, the present study adopts the traditional genus name *Mycobacterium* for clarity and consistency (Tortoli et al., 2019).

Here, we report the case of a 78-year-old woman from Guárico State, Venezuela, who presented with a chronic granulomatous lesion of the nostril persisting for more than two years. A rapidly growing mycobacterium was isolated from three independent biopsies; however, multiple diagnostic approaches—including PRA-*hsp65*, the GenoType *Mycobacterium* CM v2.0 - line probe assay (Hain Lifescience GmbH, Nehren, Germany), and partial sequencing of the *16S rRNA*, *hsp65*, and *rpoB* genes—failed to achieve species-level identification. MALDI-TOF MS (Bruker, Billerica, USA) suggested affiliation with the *M. fortuitum* group but yielded scores below the species-identification threshold. Whole-genome sequencing (WGS) using Illumina technology ultimately demonstrated that the three isolates represent a novel species, for which we propose the name *Mycobacterium venezuelense* sp. nov.

## Materials and methods

### Clinical case presentation

A 78-year-old woman with diabetes mellitus, residing in Guárico State, Venezuela, was clinically evaluated in June 2009 for a persistent lesion in her right lower nasal cavity (photographic record not provided). According to the patient, the lesion first appeared in May 2008 following a traumatic laceration of the nasal cavity with soft tissue tearing. Transient clinical improvement was observed after a short course of cefoxitin 1 g every 12 h for 6 days; however, the lesion recurred shortly after each treatment. Subsequent treatment with cefadroxil 500 mg every 24 h for 7 days, prescribed at the Dermatology Center of the Dr. Jacinto Convit Institute of Biomedicine at the Central University of Venezuela, Caracas, proved ineffective, and the infection progressively worsened. A biopsy, submitted for histopathological analysis, revealed the presence of granulomas (report not provided). The patient subsequently received empirical intravenous treatment for 4 months with linezolid 600 mg every 12 h and amikacin 500 mg every 12 h for 4 weeks, resulting in complete clinical resolution of the lesion. She was declared cured in March 2010.

### Primary mycobacterial culture in Venezuela

Because infection with a nontuberculous mycobacterium (NTM) was suspected, an initial biopsy specimen was submitted to the Tuberculosis Laboratory of the same institute for decontamination, culture, assessment of colony morphology, and species identification using PCR–restriction enzyme analysis of hsp65 (PRA-*hsp65*). The biopsy fragment was decontaminated by incubation in a 0.75% hexadecylpyridinium chloride (HPC) solution for 2 min, following the method described by Gao et al. (2005). The HPC was then removed by brief rinsing in sterile water, after which the tissue was homogenized in phosphate-buffered saline (PBS). The resulting suspension was inoculated onto Löwenstein–Jensen (LJ) medium and incubated at 37 °C. Cultures were examined for growth every 2–3 days. After 6 days, acid-fast bacteria were isolated. PCR–restriction enzyme analysis of hsp65 (PRA-*hsp65*) (Telenti et al., 1993) was used to identify the strain; however, the resulting restriction pattern was inconclusive and did not correspond to any known *Mycobacterium* species. Two additional biopsy specimens obtained subsequently yielded isolates with PRA-*hsp65* patterns identical to that of the initial strain. The three isolates were designated LTG2003-1 to LTG2003-3. The patient received empirical treatment for 4 months with linezolid and for 4 weeks with amikacin, resulting in complete clinical resolution of the lesion. She was declared cured in March 2010.

All three isolates were sent to the Laboratory of Bacteriology and Bioassays in Tuberculosis and other Mycobacteria (LBBTM), National Institute of Infectology (INI), Oswaldo Cruz Foundation (FIOCRUZ), Rio de Janeiro, Brazil, for comprehensive species identification. Analyses included phenotypic characterization, drug susceptibility testing, line probe assays, Sanger sequencing of the *16S rRNA*, *rpoB*, and *hsp65* genes, MALDI-TOF mass spectrometry, and whole-genome sequencing. At LBBTM/INI, the isolates were cataloged as LTG2003(1), LTG2003(2), and LTG2003(3) under collection code BK912/719.

### Morphological and phenotypic characterization in Brazil

Acid-fast bacilli were confirmed by Ziehl–Neelsen (ZN) staining. Colony morphology on Löwenstein–Jensen (LJ) medium, 7H10, and 7H10 + PANTA. Growth characteristics, including pigment production, temperature tolerance (30 °C, 37 °C, and 42 °C), and growth rate—were evaluated under both light and dark incubation conditions. Pigment production was assessed by exposing cultures incubated in the dark to bright white fluorescent light for 3 h, followed by an additional 18-h incubation to differentiate photochromogenic from scotochromogenic species (Forbes et al., 2018).

Classical biochemical assays were performed as described by Kent et al. (1985). These included growth on LJ medium supplemented with 5% NaCl; growth on MacConkey agar without crystal violet; citrate and iron utilization; Tween 80 hydrolysis; potassium tellurite and nitrate reduction; urea hydrolysis; and the semiquantitative catalase test.

### Identification by PCR–restriction enzyme analysis of *hsp65* (PRA)

A 441-bp fragment of the *hsp65* gene was amplified by PCR using standard primers. The PCR products were digested separately with the restriction enzymes *Bst*EII and *Hae*III, and the resulting fragments were resolved by electrophoresis on a 3% agarose gel. This approach generates species-specific banding patterns characteristic of individual *Mycobacterium* species (Telenti et al., 1993). The observed restriction patterns were compared with established PRA-*hsp65* databases and published reference tables for species identification.

### Identification by Genotype *Mycobacterium* molecular line-probe assay

Species identification was further attempted using the Genotype *Mycobacterium* CM v2.0 line-probe assay (LPA; Hain Lifescience, Nehren, Germany), which detects the *Mycobacterium tuberculosis* complex (MTBC) and 13 clinically relevant NTMs, including *M. avium*, *M. intracellulare*, *M. abscessus* complex, the *M. fortuitum* group, *M. kansasii*, *M. gordonae*, *M. xenopi*, and others. DNA was extracted from pure cultures using the GenoLyse kit (Hain Lifescience, Nehren, Germany), followed by multiplex PCR targeting the 23S rRNA gene, according to the manufacturer’s instructions. Biotinylated amplicons were hybridized to species-specific probes immobilized on nitrocellulose strips, and resulting banding patterns were visually interpreted (Lee et al., 2009; Caulfield et al., 2019).

### Molecular identification

Identification was performed by partial sequencing the *16S rRNA*, *rpoB*, and *hsp65* genes. DNA was extracted from cultured isolates using the BIO GENE K205-2 gDNA extraction kit (described later). Target gene regions were amplified using published primers and protocols specific to mycobacteria (Cooksey et al., 2004). Amplicons were purified using the commercial GFX PCR DNA and Gel Band Purification Kits (Cytiva, Massachusetts, USA) and subjected to Sanger sequencing on an ABI 3730xL sequencer, in both forward and reverse directions. The resulting sequences were compared to reference sequences in the NCBI GenBank database using the BLASTn algorithm. Species identification was based on maximum sequence similarity, applying the established thresholds of ≥99% identity for the *16S* rRNA gene and ≥97% for the *hsp65* and *rpoB* genes (Kim and Shin, 2018; Hall et al., 2003). Phylogenetic analyses were performed to support identification when BLAST results were inconclusive.

### Identification by MALDI-TOF mass spectrometry

This technique is based on the ionization of sample proteins and the subsequent separation of ions by mass, generating a mass spectral profile that is compared against a reference protein database (Oliva et al., 2021). MALDI-TOF MS was performed using the Bruker Biotyper system (Bruker Daltonics, Karlsruhe, Germany) with the Mycobacteria RUO Library v4.0 (Bruker Daltonics, Karlsruhe, Germany) for spectral matching. Colonies were subjected to ethanol-formic acid extraction following the manufacturer’s protocol (Genc et al., 2018). Spectral acquisition was carried out using a Microflex LT/SH system, and analysis was performed with Bruker Biotyper software. Identification scores ≥2.0 were considered reliable for species-level identification (Costa-Alcalde et al., 2019).

### Drug susceptibility testing

DST of the RGM was performed using the commercial Sensititre™ RAPMYCO^1^ susceptibility test plate assay (Thermo Fisher Scientific, Waltham, USA), which includes 15 antibiotics: amikacin, cefoxitin, moxifloxacin, ciprofloxacin, clarithromycin, doxycycline, imipenem, linezolid, trimethoprim-sulfamethoxazole, tobramycin, cefepime, amoxicillin/clavulanic acid, ceftriaxone, minocycline and tigecycline (Wetzstein et al., 2020). All tests were performed in triplicate and plates were incubated at 30 °C for three to 7 days. Test interpretation was according to the Clinical and Laboratory Standards Institute (CLSI) M24S, which recommends *Mycobacterium peregrinum* ATCC® 27853 and *Staphylococcus aureus* ATCC ® 29213 as microorganisms for quality control, using breakpoints described for RGM (CLSI, 2023; Bhalla et al., 2018; Woods et al., 2011) and published data (CLSI, 2018; Brown-Elliott et al., 2016).

#### DNA extraction, purification, and whole-genome sequencing

For DNA preparation from the three isolates, bacterial cells from three loops were resuspended in 200 μL of 1X Tris-EDTA (TE) buffer. After vigorous vortexing for 5 min, the suspension was incubated at 95 °C for 30 min for bacterial inactivation. Genomic DNA was then extracted using the BIO GENE gDNA Extraction Kit K205-2 (Bioclin, Belo Horizonte, Brazil) according to the manufacturer’s instructions. DNA quality and concentration were assessed using the Qubit® dsDNA High Sensitivity (HS) Assay Kit (Thermo Fisher Scientific, Waltham, USA). Whole-genome sequencing (WGS) was performed on an Illumina MiSeq platform (Illumina, San Diego, CA, USA). Sequencing libraries were prepared using the Nextera XT Kit (Illumina), generating 2 × 150 bp paired-end reads.

### Genomic analysis and initial specie identification

WGS data were analyzed using the Bacass v2.3.1 pipeline, implemented in Nextflow DSL2 with Docker/Singularity containers for reproducibility. Genome assembly was carried out with Unicycler v0.4.8 and Kraken2 (standard 8 GB database, April 2025) was used to assess contamination, and results were visualized using Krona plots (Wick et al., 2017; Wood et al., 2019). CheckM v1.2.3 was used to evaluate genome completeness (Gurevich et al., 2013; Parks et al., 2015). To determine taxonomic placement, average nucleotide identity (ANI; using the NCBI Prokaryotic Genome Annotation Pipeline (PGAP) taxonomy verification tool) and digital DNA–DNA hybridization (dDDH; using the automated Type Strain Genome Server (TYGS) platform, Meier-Kolthoff and Göker, 2019; Meier-Kolthoff et al., 2014) values were calculated by comparing the genome with the genomes of *Mycobacterium* species present in GenBank. Species assignment was based on cutoffs of ANI > 95% and dDDH > 70%; subspecies assignment used ANI ≤ 97% and dDDH ≤ 80% (Iversen et al., 2025). Annotation was performed using both the NCBI Prokaryotic Genome Annotation Pipeline (PGAP) and the RAST server, which provide structural and functional annotation for prokaryotic genomes.

### Phylogenetic analysis

Phylogenetic relationships were inferred using Mashtree, which combines the min-hash algorithm from the Mash tool (Ondov et al., 2011) with the neighbor-joining (NJ) algorithm from QuickTree (Howe et al., 2002). A Mash distance matrix was computed and used to construct a dendrogram with default parameters. The resulting tree was visualized and annotated using iTOL (Interactive Tree of Life) (Meier-Kolthoff et al., 2013; Kreft et al., 2017). Species labels corresponding to *M. wolinskyi* genomes (GCF accessions from GenBank) were included for comparison with the genomes of the three LTG2003 isolates (1, 2, and 3).

### Taxonomic identification

Phylogenetic trees based on ANI values were constructed to complement the Mash-based analysis, providing further resolution of genomic relatedness among the isolates and reference strains. WGS data was uploaded to the Type (Strain) Genome Server (TYGS) platform^2^ for detailed genome-based taxonomic analysis (Meier-Kolthoff and Göker, 2019; Meier-Kolthoff et al., 2022). TYGS calculates ANI and dDDH values and constructs phylogenomic trees based on WGS comparisons. This analysis was supplemented with current nomenclature and taxonomic information from the TYGS’s linked database and List of Prokaryotic names with Standing in Nomenclature (LPSN).^3^ The results from TYGS, received on 31st of March of 2025, provided further resolution for species delineation and taxonomic placement (Turenne, 2019).

## Results

### Phenotypic and biochemical characterization LTG2003(1–3)

**Figure 1:**
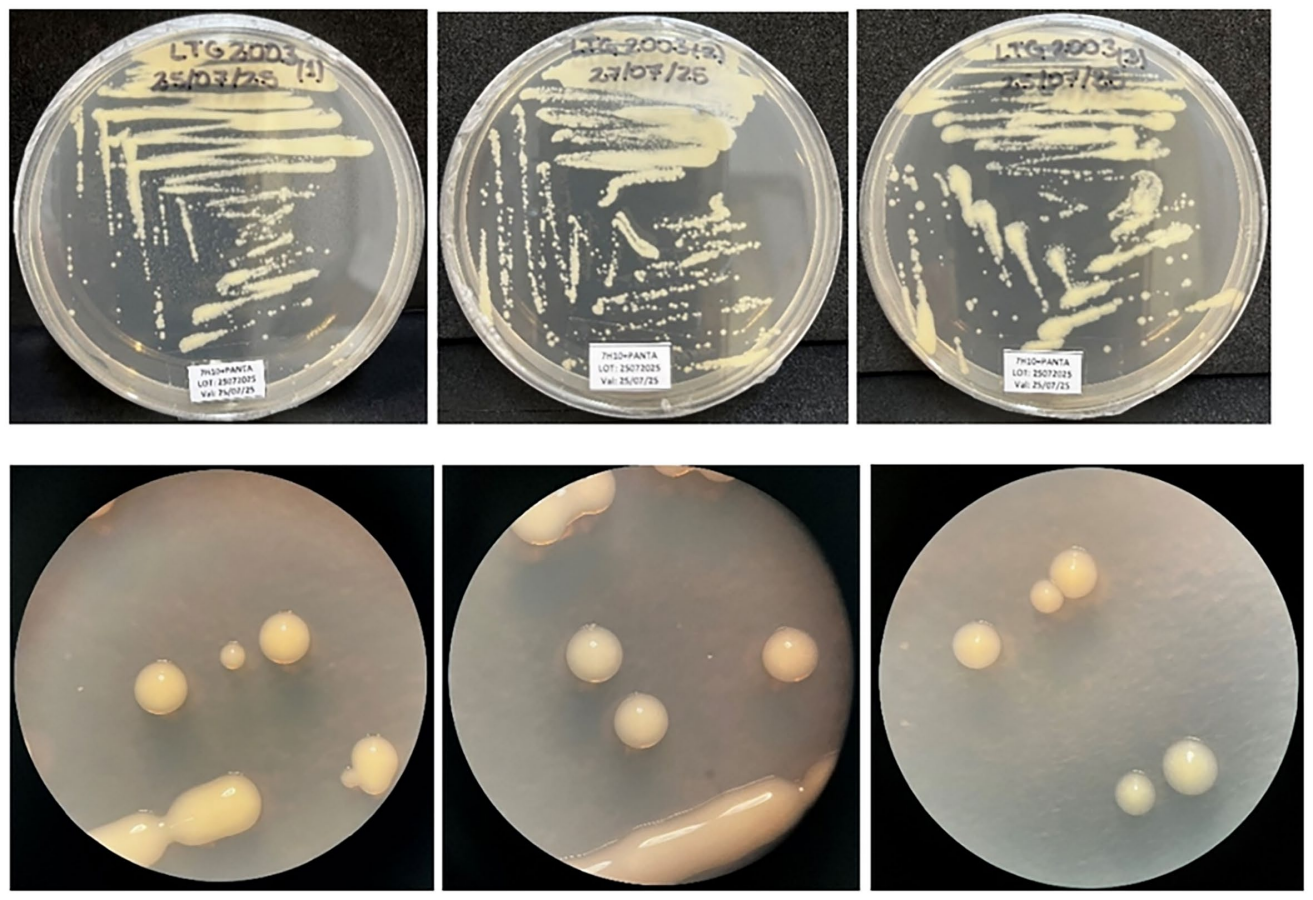
(A) Cultures of isolates LTG2003(1–3) grown on 7H10 + PANTA medium (upper panel). (B) Colonies from each culture, captured using a stereoscopic microscope at 1.6X magnification (lower panel). Cultures of the isolates LTG2003(1–3) on 7H10 + PANTA medium (upper line), and image of the colonies of each culture, captured with a stereoscopic microscope at 1.6X (lower line).

All three isolates, LTG2003(1–3) (later renamed BK912/719(1–3) at the LBBTM, INI-FIOCRUZ), formed smooth, creamy, non-pigmented colonies that grew at 30 °C, 37 °C, and 42 °C after 5 days of incubation on either L-J or 7H10 + PANTA medium, with no differences in morphology (Figure 1 and Supplementary Figure 1). Colonies on 7H10 medium were photographed after 5 days, highlighting their non-pigmented appearance. As the three isolates originated from separate biopsies of the same lesion and exhibited identical colonial morphology, each was independently subjected to biochemical testing and molecular analyses.

All isolates tested positive for urea hydrolysis, tellurite reduction (on days 7 and 10), arylsulfatase activity (on day 15), and semi-quantitative catalase. They also showed growth on Löwenstein-Jensen medium supplemented with NaCl, iron, and citrate (after 3 weeks). Weak positivity was observed for Tween 80 hydrolysis, and on MacConkey agar, weak positivity appeared on day 11 for the first two isolates and complete positivity for the last isolate. Negative results were specific for the pigmentation test, indicating an absence of pigment production, classifying the isolates as non-chromogenic whites (Group IV - Runyon classification). When compared with the identification scheme proposed by Fernández de Vega F. et al. (2005), the biochemical profile most closely resembled that of *M. mageritense*, *M. smegmatis*, and *M. wolinskyi*. However, *M. wolinskyi* typically tests negative for arylsulfatase after 3 days, whereas our isolates showed weak positivity at 3 days and full positivity at 15 days for this test. Table 1 presents a summary of the phenotypic and biochemical results.

**Table 1:** Phenotypic and biochemical characteristics of the three isolates (Kent et al., 1985).

Sample codes	Urea		Tween 80 Hydrolysis		Tellurite Reduction		Arylsulfatase		Semi-quantitative catalase	Growth in MacConkey		Growth in NaCl		Iron Uptake		Citrate		Growth rate		Pigmentation		
	18 h	3d	5d	10d	7d	10d	3d	15d	15d	5d	11d	15d	3S	15d	3S	15d	3S	7d	15d	7d	15d	After exposed to light
LTG2003 (1)	POS	POS	POS-weak	POS-weak	POS	POS	POS-weak	POS	POS	NEG	POS-weak	POS	POS	POS	POS	–	POS	<7d	–	NEG	NEG	White-Non-chromogenic (Group IV)
LTG2003 (2)	POS	POS	POS-weak	POS-weak	POS	POS	POS-weak	POS	POS	NEG	POS-weak	POS	POS	POS	POS	–	POS	<7d	–	NEG	NEG	White-Non-chromogenic (Group IV)
LTG2003 (3)	POS	POS	POS-weak	POS-weak	POS	POS	POS-weak	POS	POS	NEG	POS	POS	POS	POS	POS	–	POS	<7d	–	NEG	NEG	White-Non-chromogenic (Group IV)

### Identification by molecular techniques

**Figure 2:**
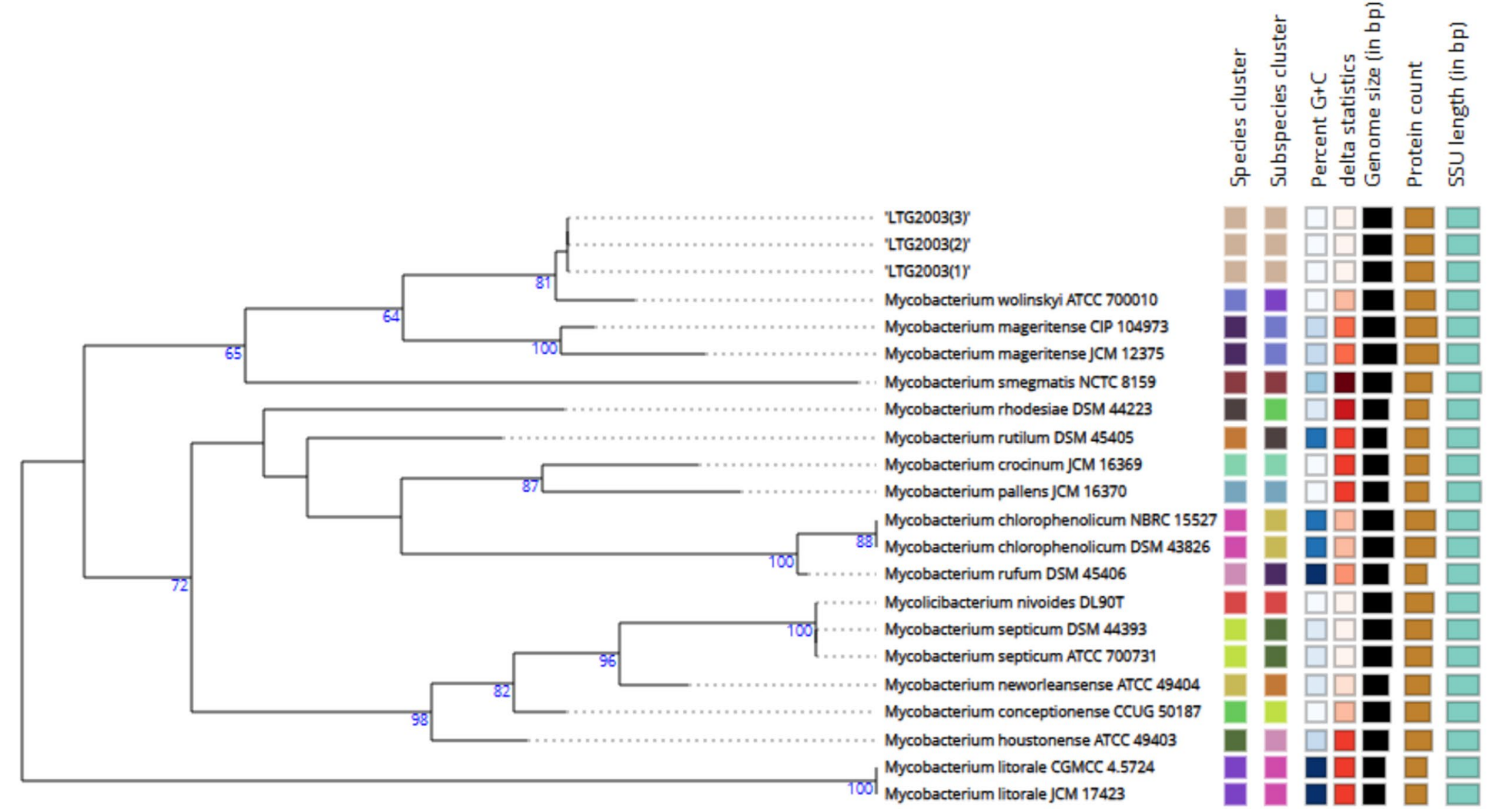
Phylogenetic tree inferred from GBDP distances based on *16S rRNA* gene sequences of LTG2003 (1–3), using FastME 2.1.6.1 (Lefort et al., 2015). Branch lengths are scaled according to the GBDP distance formula d5. Numbers above branches represent GBDP pseudo-bootstrap support values >60% from 100 replicates; the average branch support was 71.3%. The tree was rooted at the midpoint (Farris, 1972).

**Figure 3:**
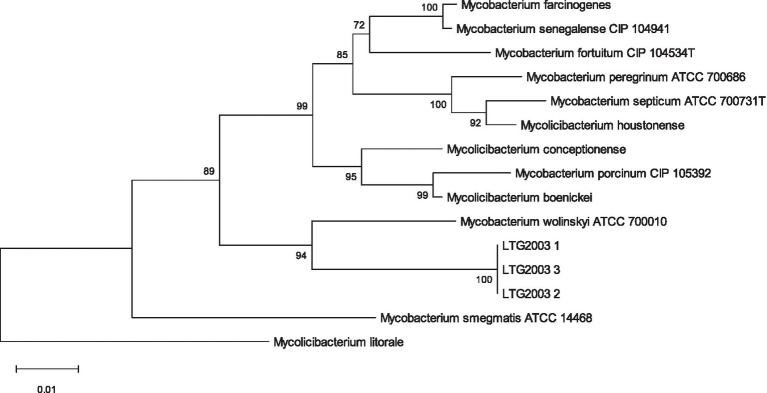
Phylogenetic tree of the LTG2003 strains (1, 2, and 3) using neighborhood-joining statistical methods and the Kimura two-parameter nucleotide substitution model for the *rpoB* gene. *M. litorale* was used as an outgroup to root the tree. A comparative analysis was performed using partial sequencing data from LTG2003 strains and FASTA sequences of the *rpoB* gene available in GenBank from the species with the greatest ANI similarity to the study samples.

**Figure 4:**
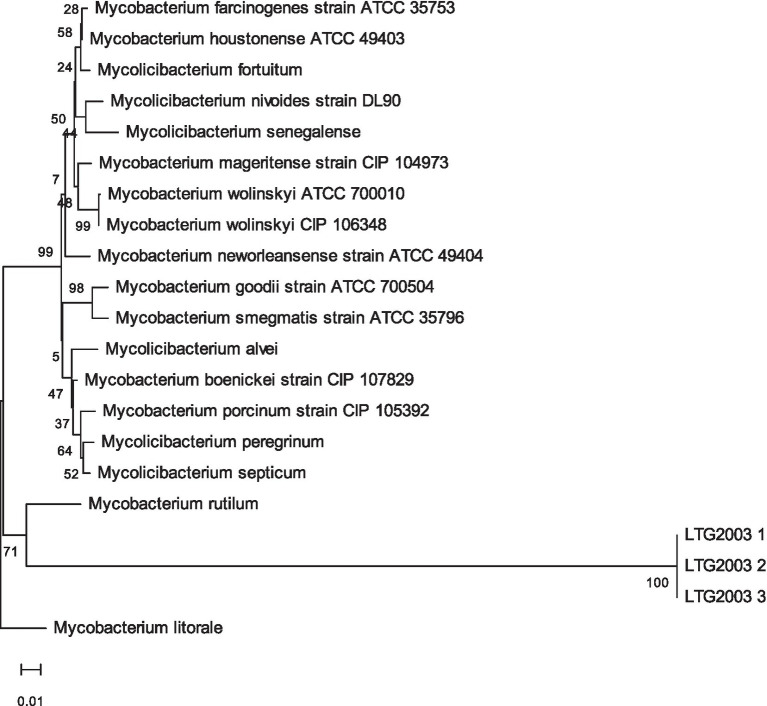
Phylogenetic tree of LTG2003 strains (1–3) using neighborhood-joining statistical methods and the Kimura two-parameter nucleotide substitution model for the *hsp65* gene. *Mycobacterium litorale* was used as an outgroup to root the tree. A comparative analysis was performed using partial sequencing data from the LTG2003 strains and FASTA sequences of the *hsp65* gene available in GenBank from the species with the greatest ANI similarity to the study samples.

PRA-*hsp65* method yielded restriction patterns (*BstE*II: 231/131/79-bp; *Hae*III: 139/117/58/51/40/36-bp), that did not correspond to any previously described species (Telenti et al., 1993. Devallois et al., 1997). Supplementary Figure 2 presents the results of the Genotype *Mycobacterium* CM assay, which classified the three isolates within the *M. fortuitum* group. MALDI-TOF MS produced low-confidence genus-level scores ranging from 1.63 to 1.82, all suggesting *M. wolinskyi* but falling below the ≥2.0 threshold required for species assignment (Table 2). Partial Sanger sequencing of the *16S rRNA, rpoB,* and *hsp65* genes also failed to definitively identify the isolates. Phylogenetic trees for each marker (Figures 2–4), constructed using the neighbor-joining method with 1,000 bootstrap replicates, consistently placed the isolates within a clade closely related to *M. wolinskyi*, however, bootstrap support was insufficient for definitive species-level resolution. As expected, the *16S rRNA* gene lacked discriminatory power. While *rpoB* and *hsp65* provided greater resolution, their sequences remained inconclusive for assignment to a recognized species (GenBank accession numbers SUB15741956, SUB15743129, and SUB15743112, respectively). These findings prompted whole-genome sequencing.

**Table 2:** Results of MALDI-TOF mass spectrometry analysis of isolates LTG2003(1), (2), and (3), performed at the Bacteriology and Bioassays Reference Laboratory, National Institute of Infectology – FIOCRUZ.

Sample codes	Identification – MALDI TOF	Score
LTG2003(1)	*Mycobacterium wolinskyi*	1.63
LTG2003(2)	*Mycobacterium wolinskyi*	1.82
LTG2003(3)	*Mycobacterium wolinskyi*	1.69

Score interpretation: 2.00–3.00: High-confidence species-level identification. 1.70–1.99: Low-confidence (genus-level) identification. 0.00–1.69: Identification not reliable or not possible.

### Antibiotic susceptibility of the LTG2003 strains

Accurate species identification, DST, are essential for selecting effective therapies against mycobacterial infections. In this case, MIC testing was performed on the three isolates in triplicate. The isolates were resistant in each test (of the triplicate) to ciprofloxacin, clarithromycin, sulfamethoxazole, trimethoprim, and tobramycin; showed intermediate susceptibility to cefoxitin, moxifloxacin, and imipenem; and remained susceptible to amikacin and doxycycline. The MIC value for tigecycline was 0.12 μg/mL, for cefepime was >32 μg/mL, for amoxicillin/clavulanic acid was 16/8 μg/mL, for ceftriaxone was >64 μg/mL, and for minocycline was 1 μg/mL for each sample in each triplicate (Table 3).

**Table 3:** In vitro antibiotic susceptibility profile of the *Mycobacterium* isolates as determined with the Sensititre™ RAPMYCO susceptibility test plate assay (Thermo Fisher Scientific, Waltham).

Antibiotics	LTG2003_(1)_		LTG2003_(2)_		LTG2003_(3)_		MIC quality control ranges (μg/mL)	
	MIC (μg/mL)	MIC categories	MIC (μg/mL)	MIC categories	MIC (μg/mL)	MIC categories	*M. peregrinum* ATCC® 700,686	*S. aureus* ATCC ® 29,213
Amikacin	1	S	1	S	1	S	≤1–4	1–4
Cefoxitin	32	I	32	I	32	I	4–32	1–4
Moxifloxacin	2	I	2	I	2	I	≤0.06–0.25	0.016–0.12
Ciprofloxacin	4	R	4	R	4	R	≤0.12–0.5	0.12–0.5
Clarithromycin	16	R	16	R	16	R	≤0.06–0.5	0.12–0.5
Doxycycline	0.12	S	0.12	S	0.12	S	0.12–0.5	0.12–0.5
Imipenem	8	I	8	I	8	I	2–16	0.016–0.06
Linezolid	1	S	1	S	1	S	1–8	1–4
Trimethoprim-sulfamethoxazole	8/152	R	8/152	R	8/152	R	≤0.25/4.8–2/38	≤0.5/9.5
Tobramycin	16	R	16	R	16	R	2–8	0.12–1
Tigecycline	0.12	–	0.12	–	0.12	–	0.03–0.25	0.03–0.25
Cefepime	>32	–	>32	–	>32	–	–	^a^
Amoxicillin/clavulanic acid	16/8	–	16/8	–	16/8	–	–	0.12/0.06–0.5/0.25
Ceftriaxone	>64	–	>64	–	>64	–	–	1–8
Minocycline	1	–	1	–	1	–	0.12–0.5	0.06–0.5

a No broth microdilution quality control range when testing for fast-growing mycobacteria in cation-adjusted Mueller-Hinton broth without OADC.

### Whole genome sequencing (WGS)

WGS was employed for more detailed characterization of the isolates. Comparative genomic analyses, including phylogenetic reconstruction and genome-wide similarity searches, revealed that the isolates did not belong to any known NTM species. Consequently, we propose the name *Mycobacterium venezuelense* sp. nov. for this previously undescribed, rapidly growing mycobacterium. The genome sequence has been deposited in the NCBI DDBJ/ENA/GenBank database with accession number JBONDW000000000 (provisional name: *Mycobacterium* sp. LTG2003-1), and the record was published on February 5, 2026. As shown in Table 4, the three genomes exhibited nearly identical assembly metrics, consistent with their status as a technical triplicate. The three assemblies were of high-quality, featuring low contig counts (max: 142), high N50 values (123,307–324,789-bp), 100% completeness, and minimal contamination (1.68%). Genomic characteristics were also uniform, with GC content between 66.22–66.24% and similar counts of coding sequences (CDSs), rRNA, and rRNA operons.

**Table 4:** Genome assembly and annotation quality metrics.

Bioinformatics tools	Spades	Spades	Spades	CheckM - Bacass	CheckM - Bacass	Spades	Prokka	Prokka	Prokka
ID	Genome size	Contigs	N50 (Kb)	Completeness (%)	Contamination (%)	G + C (%)	CDSs	rRNA operons	tRNAs operons
LTG2003(1)	7,073,873	64	298.790	100.00	1.68	66.23	6,748	2	56
LTG2003(2)	7,069,248	142	123.307	100.00	1.68	66.24	6,758	2	56
LTG2003(3)	7,078,342	70	324.789	100.00	1.68	66.22	6,754	2	56
Control: H37Rv	4,365,222	94	115.351	–	–	65.59	–	–	–

Taxonomic profiling using the Kraken bioinformatics tool assigned a majority of reads to the novel *Mycobacterium* sp. A minority of reads were classified as belonging to other mycobacterial species, including *M. goodii* (5%), *M. margeritense* (4%), *M. fortuitum* (2%) and *M. litorale* (1%) (Supplementary Figures 3–5).

Using taxonomic characterization parameters based on ANI and dDDH, we observed (Tables 5, 6) that the three isolates showed ANI values ranging from 91.052 to 91.054 with *M. wolinskyi* (GCA_002101965.1, ASM210196v1) and dDDH values of 42.7–42.8% with *M. wolinskyi* ATCC 700010.

**Table 5:** Average nucleotide identity (ANI) results obtained using the NCBI-developed automated prokaryotic genome annotation pipeline PGAP.

ID	ANI	(Coverages)	NewSeq	CntmSeq	Assembly	Organism (assembly_accession, assembly_name)
LTG2003 (1)	91.053	(84.6 78.8)	4,646,483	4,646,483	4,389,068	*Mycolicibacterium wolinskyi* (GCA_002101965.1, ASM210196v1)
	91.050	(84.6 78.8)	1,263,474	1,263,474	36,891,498	*Mycolicibacterium wolinskyi* (GCA_025822475.1, ASM2582247v1)
	82.821	(38.6 42.3)	182	182	5,154,288	*Mycolicibacterium houstonense* (GCA_900078665.2, PRJEB13221)
LTG2003 (2)	91.054	(84.6 78.8)	4,886,841	4,886,841	4,389,068	*Mycolicibacterium wolinskyi* (GCA_002101965.1, ASM210196v1)
	91.051	(84.6 78.8)	1,011,545	1,011,545	36,891,498	*Mycolicibacterium wolinskyi* (GCA_025822475.1, ASM2582247v1)
	82.833	(38.5 42.2)	174	174	5,154,288	*Mycolicibacterium houstonense* (GCA_900078665.2, PRJEB13221)
LTG2003 (3)	91.052	(84.6 78.8)	4,774,367	4,774,367	4,389,068	*Mycolicibacterium wolinskyi* (GCA_002101965.1, ASM210196v1)
	91.048	(84.6 78.8)	1,134,627	1,134,627	36,891,498	*Mycolicibacterium wolinskyi* (GCA_025822475.1, ASM2582247v1)
	82.828	(38.6 42.3)	3,553	3,553	5,154,288	*Mycolicibacterium houstonense* (GCA_900078665.2, PRJEB13221)

This analysis describes genome similarity and contamination metrics calculated against type strain assemblies. ANI (Average Nucleotide Identity): The average nucleotide identity between the query assembly and the type strain assembly. Coverages: The query and subject coverage values derived from the ANI calculation. NewSeq: The number of bases in the query assembly that were best assigned to the type strain. CntmSeq: The proportion of the NewSeq value flagged as potential contamination. Assembly: The GenBank assembly accession for the type strain used in the comparison. Organism: The name and assembly details (Accession, Name) of the type strain.

**Table 6:** Pairwise comparisons of LTG2003 (1, 2, and 3) - genomes versus type strain genomes from the NCBI database used by the TYGS Taxonomic Genomic Analysis Server.

Query strain	Subject strain	dDDH (d0, in %)	C.I. (d0, in %)	dDDH (d4, in %)	C.I. (d4, in %)	dDDH (d6, in %)	C.I. (d6, in %)	G + C content difference (in %)
LTG2003 (1)	*Mycobacterium wolinskyi* ATCC 700010	71.1	[67.2–74.8]	42,7	[40.2–45.3]	65,6	[62.3–68.9]	0,18
LTG2003 (2)	*Mycobacterium wolinskyi* ATCC 700010	71.1	[67.1–74.7]	42,8	[40.2–45.3]	65,6	[62.2–68.8]	0,18
LTG2003 (3)	*Mycobacterium wolinskyi* ATCC 700010	71.1	[67.1–74.7]	42,7	[40.2–45.3]	65,6	[62.2–68.8]	0,18

**Figure 5:**
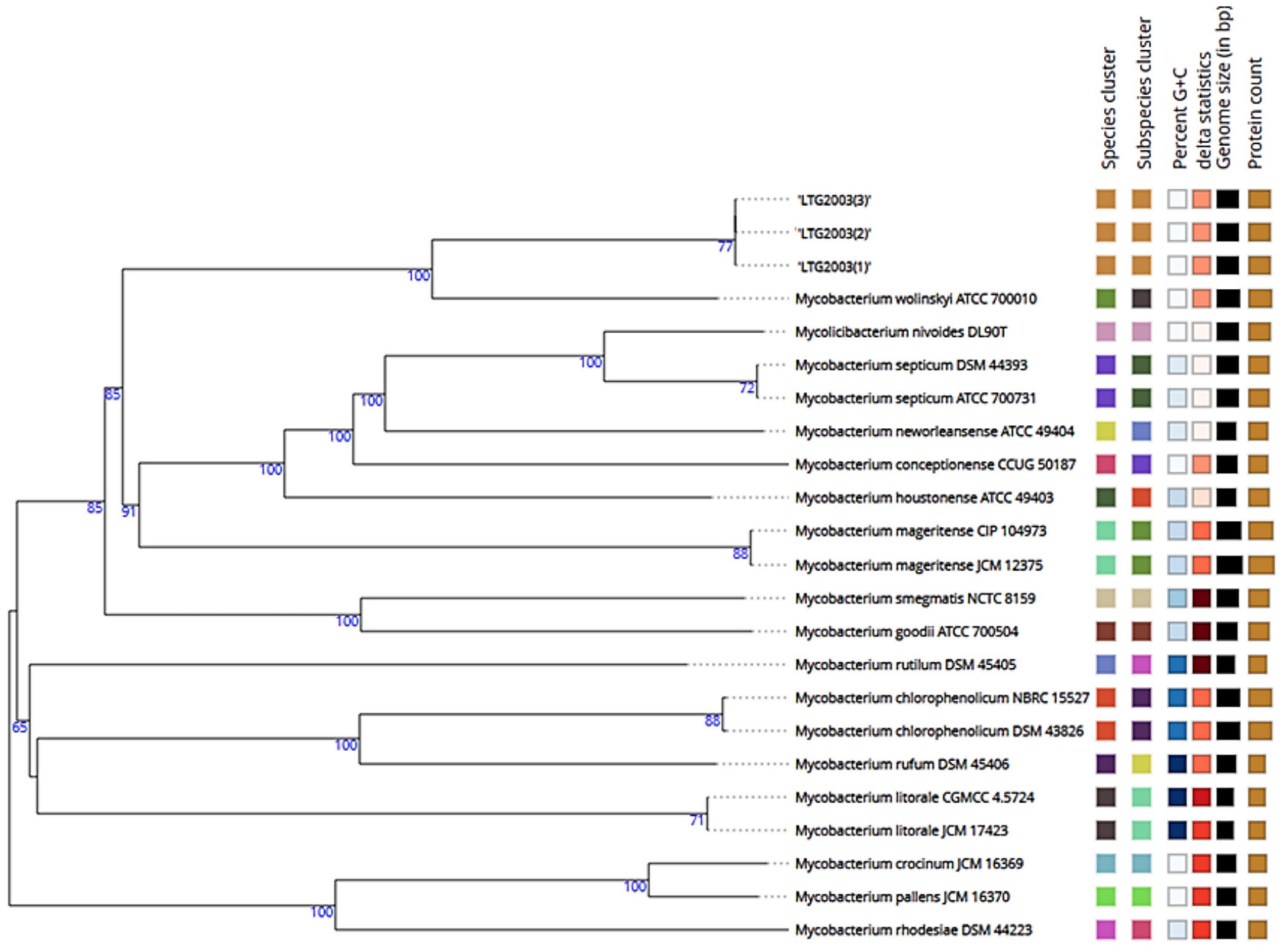
Phylogenetic tree inferred with FastME 2.1.6.1 from GBDP distances calculated using the genomic sequences of isolates LTG2003(1–3). Branch lengths are scaled according to the GBDP distance formula d5. Numbers above branches represent GBDP pseudo-bootstrap support values >60% based on 100 replications, with an average branch support of 84.6%. The tree was rooted at the midpoint (Farris, 1972; Lefort et al., 2015).

Using the TYGS platform, we generated the phylogenetic tree shown in Figure 5, based on GBDP distances calculated from the genomic sequences. The isolates LTG2003(1), (2), and (3) clustered as a distinct species, separate from the closest reference strain, *Mycobacterium wolinskyi* ATCC 700010, which occupies a different branch of the tree.

**Figure 6:**
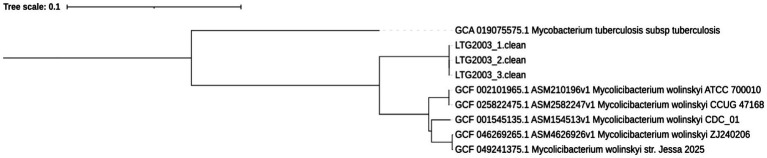
Phylogenetic tree generated using the bioinformatics tools Mashtree and iTOL, comparing the genomes of isolates LTG2003(1–3) with all *Mycolicibacterium wolinskyi* genomes currently available in the NCBI database.

In addition, a comparative phylogenetic tree including all *M. wolinskyi* genomes currently available in the NCBI GenBank database, together with the three genomes obtained in this study, was constructed using the bioinformatics tools Mashtree and iTOL. *M. tuberculosis* subsp. *tuberculosis* (GCA_019075575.1) was used as an outgroup to root the tree. In this analysis, the isolates LTG2003(1), (2), and (3) formed a separate branch from the five known *M. wolinskyi* genomes: ATCC 700010, CCUG 47168, CDC_01, ZJ240206, and strain Jessa 2025 (the latter recently added to the NCBI database) (Figure 6).

**Figure 7:**
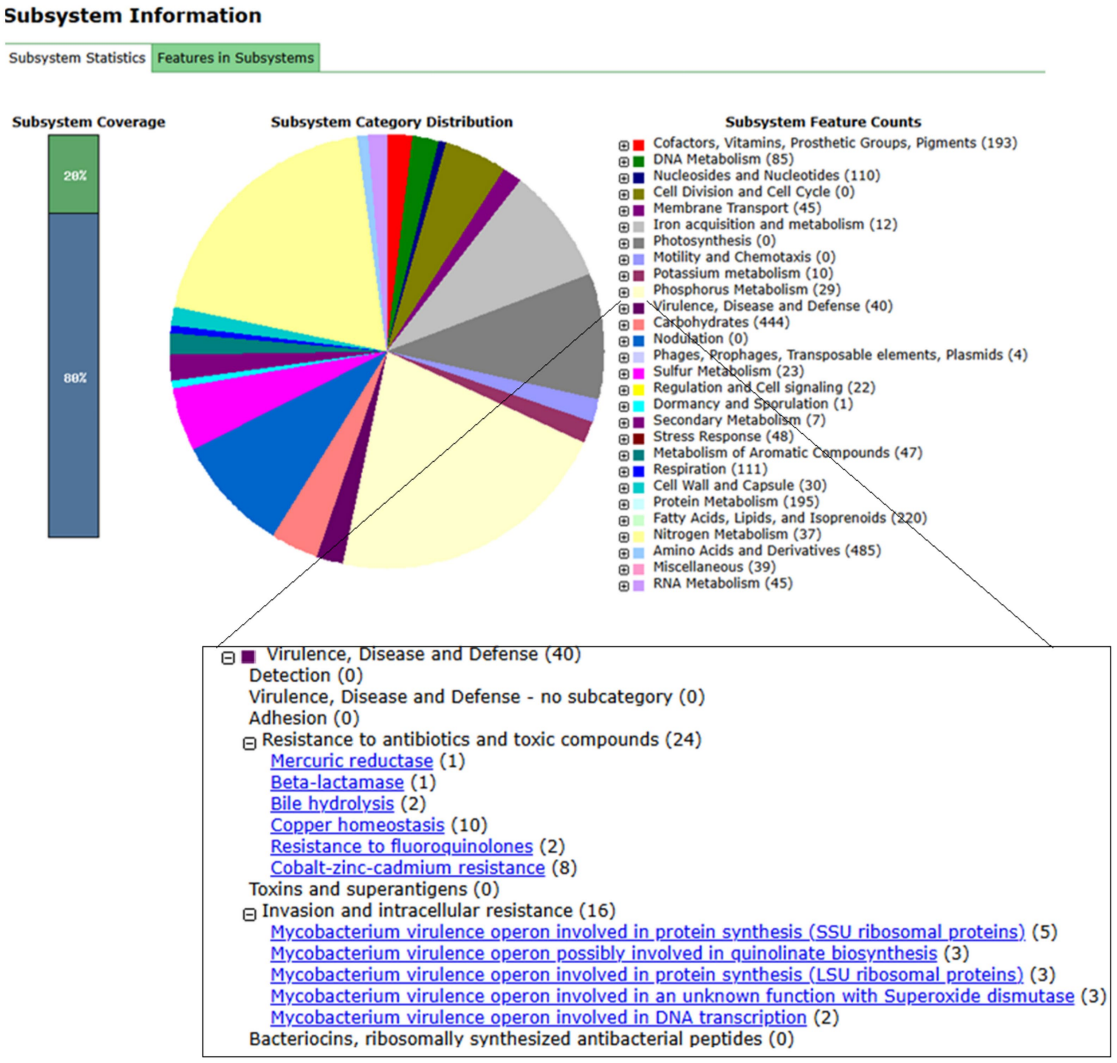
The genome of strain LTG2003(1) was annotated using the RAST server. Key assembly statistics and annotation features are summarized below, followed by the subsystem categorization, with a specific focus on genes related to virulence, disease, and defense (bottom panel). Genome assembly & general features: size: 7,005,557 bp; GC content: 66.2%; contigs: 89 (containing protein encoding genes, PEGs); assembly quality (N50/L50): The N50 statistic is 298,684 bp, with an L50 of 9. This indicates that half of the total assembly length is contained in contigs of at least ~298.7 kb, and this threshold is reached by just nine of the largest contigs, reflecting good assembly contiguity. RAST annotation summary: Coding sequences (CDS): 6,899; RNAs: 51. Subsystems: 317 distinct functional categories were identified. The resulting subsystem distribution is shown in the figure, highlighting the complement of genes associated with virulence, disease, and defense mechanisms.

For genomic annotation, only one of the three isolates, LTG2003-1, was deposited in Genebank. Annotation was performed using the RAST server, which predicted a total of 6,899 coding sequences (CDSs) organized into 317 subsystems with a 20% coverage (Figure 7). The largest subsystem was that for amino acids and derivatives (485 genes), followed by carbohydrates (444), fatty acids, lipids and isoprenoids (220), protein metabolism (195), cofactors, vitamins, prosthetic groups and pigments (193), respiration (111), nucleosides and nucleotides (110), and DNA metabolism (85).

The virulence, disease, and defense subsystem comprised 40 genes, including 16 genes related to intracellular invasion and resistance. These included *Mycobacterium* virulence operons involved in the synthesis of SSU ribosomal proteins (5 genes), LSU ribosomal proteins (3), quinolinate biosynthesis (3), DNA transcription (2), and superoxide dismutase of unknown function (3). The remaining 24 genes were associated with antibiotic and toxic compound resistance, including *β*-lactamase (1), mercuric reductase (1), bile hydrolysis (2), cobalt–zinc–cadmium resistance (8), fluoroquinolone resistance (2), and copper homeostasis (10).

The new strain [LTG2003(1)] was deposited under code or isolate number: P7483 in the Pathogenic Bacteria Collection of the National Institute for Quality Control in Health, Oswaldo Cruz Foundation. This collection is registered in the WDCM and managed by the WFCC under number 1312. A duplicate of the strain was also deposited in the National Collection of Bacterial Strains and Cell Cultures (WDCM CDBB-500) at the Centro de Investigación y de Estudios Avanzados del IPN (CINVESTAV), Mexico. At present, no accession number has been assigned to this deposit; however, we expect it to be available by the time of publication.

## Discussion and conclusions

### Proposal of *Mycobacterium venezuelense* sp. nov., a novel species related to *Mycobacterium wolinskyi*

We describe a new NTM species, closely related to *M. wolinskyi*. First identified in 1999, *M. wolinskyi* is a RGM (Runyon type IV) commonly found in soil and water and belonging to the *M. smegmatis* group, which is frequently associated with skin, soft tissue, and post-operative infections (Brown et al., 1999; Chen et al., 2008; Ohno et al., 2008; Ariza-Heredia et al., 2011). Since its discovery, approximately 30 clinical cases have been reported, often involving prosthetic implants, cardiovascular surgery, and cosmetic procedures (Pulcini et al., 2006; Jeong et al., 2012; Yoo et al., 2013; Karakala et al., 2013; Santos Lima et al., 2013; Lee et al., 2015).

Diagnosis of soft tissue infections relies on culture, as conventional AFB staining methods lack species-level specificity. While our initial culture confirmed the presence of acid-fast bacilli, partial sequencing of the *16S rRNA*, *rpoB*, and *hsp65* genes yielded inconclusive results, failing to definitively identify the isolates as *M. wolinskyi* or any single known species (Figures 2–4). This prompted whole-genome sequencing of the three samples.

Comparative genomic analysis of the three isolates revealed a significant taxonomic divergence from the genomes from other known species. The ANI values against the genome of the *M. wolinskyi* type strain (ATCC 700010/GCA_002101965.1) was 91.052–91.054%, and dDDH values were 42.7–42.8% (Figure 6). These results are far below the established species demarcation thresholds (ANI > 95–96%; dDDH > 70%), providing conclusive evidence that our isolates represent a novel species, for which we propose the name *Mycobacterium venezuelense* sp. nov. The genome of the proposed type strain LTG2003(1) has been deposited in GenBank under accession number JBONDW000000000.

The phenotypic and antimicrobial susceptibility profile of *M. venezuelense* sp. nov. was very similarto that of *M. wolinskyi*, showing sensitivity to amikacin, doxycycline, and linezolid, and resistance to ciprofloxacin, clarithromycin, and tobramycin (Table 3). A tigecycline MIC of 0.12 μg/mL was also recorded (Ella et al., 2011). Genomic annotation via the RAST platform provided a basis for these observations, identifying genes associated with resistance to antibiotics and toxic compounds. These included genes for a metal-dependent hydrolase of the *β*-lactamase superfamily III and for fluoroquinolone resistance (DNA gyrase subunit B), correlating with the observed intermediate resistance to imipenem and resistance to ciprofloxacin (Supplementary Table 1).

This study confirms the contribution of genomic sequencing for the accurate classification of NTMs. Our findings demonstrate that novel pathogenic species may be masked by their close phenotypic and genetic relationship to known species. The correct identification of *M. venezuelense* sp. nov., as well as other recently discovered mycobacterial species, such as *Mycobacterium wendilense* sp. nov., *Mycobacterium burgundiense* sp. nov., *Mycobacterium kokjensenii* sp. nov., and *Mycobacterium holstebronense* sp. nov. (Iversen et al., 2025), is crucial for rapid diagnosis, targeted treatment, and improved patient care, preventing the high morbidity associated with unresolved NTM infections.

### Description of the new species

*Mycobacterium venezuelense* (ve.ne.zu.e.len′se. N.L. neut. Adj. venezuelense, referring to Venezuela, the country from which the type strain was isolated) are acid-fast bacilli. Colonies are non-pigmented and grow rapidly, with visible growth observed within 7 days on Löwenstein–Jensen and Middlebrook 7H10 media at 30–37 °C. Phylogenetic analysis based on the *16S rRNA* gene sequence, together with partial sequences of *rpoB* and *hsp65*, demonstrates that the species is distinct from all other recognized members of the genus *Mycobacterium*. These sequences have been deposited in GenBank under accession numbers SUB15741956, (*16S* rRNA), SUB15743129 (*rpoB*), and SUB15743112 (*hsp65*).

The type strain, LT2003-1, was isolated from a biopsy specimen obtained from a patient with an NTM-associated soft tissue infection in Caracas, Venezuela. The strain is susceptible to amikacin, doxycycline, and linezolid, and resistant to ciprofloxacin, clarithromycin, and tobramycin; the MICs for drugs without a CLSI-standardized breakpoint were: 0.12 μg/mL for tigecycline, >32 μg/mL for cefepime, 16/8 μg/mL for amoxicillin/clavulanic acid, >64 μg/mL for ceftriaxone, and 1 μg/mL for minocycline. The genome of strain LT2003-1 consists of a single circular chromosome of 7,005,557-bp, with a DNA G + C content of 66.2% and 6,899 predicted coding sequences.

## Data Availability

The datasets presented in this study can be found in online repositories. The names of the repository/repositories and accession number(s) can be found in the article/ Supplementary material.

## Footnotes

1
https://www.thermofisher.com/order/catalog/product/RAPMYCOI?SID=srch-srp-RAPMYCOI#/RAPMYCOI?SID=srch-srp-RAPMYCOI

2
https://tygs.dsmz.de

3
https://lpsn.dsmz.de
