# Supplementary material for: Description and complete genome sequence of Mycobacterium venezuelense sp. nov., a rapidly growing species recovered from a soft tissue infection
Source: Front Microbiol. 2026 Apr 2;17:1780266. doi: 10.3389/fmicb.2026.1780266 (PMC13083205; doi:10.3389/fmicb.2026.1780266)
Supplement: Supplementary file 1 [file Data_Sheet_1.pdf]

Supplementary Figures

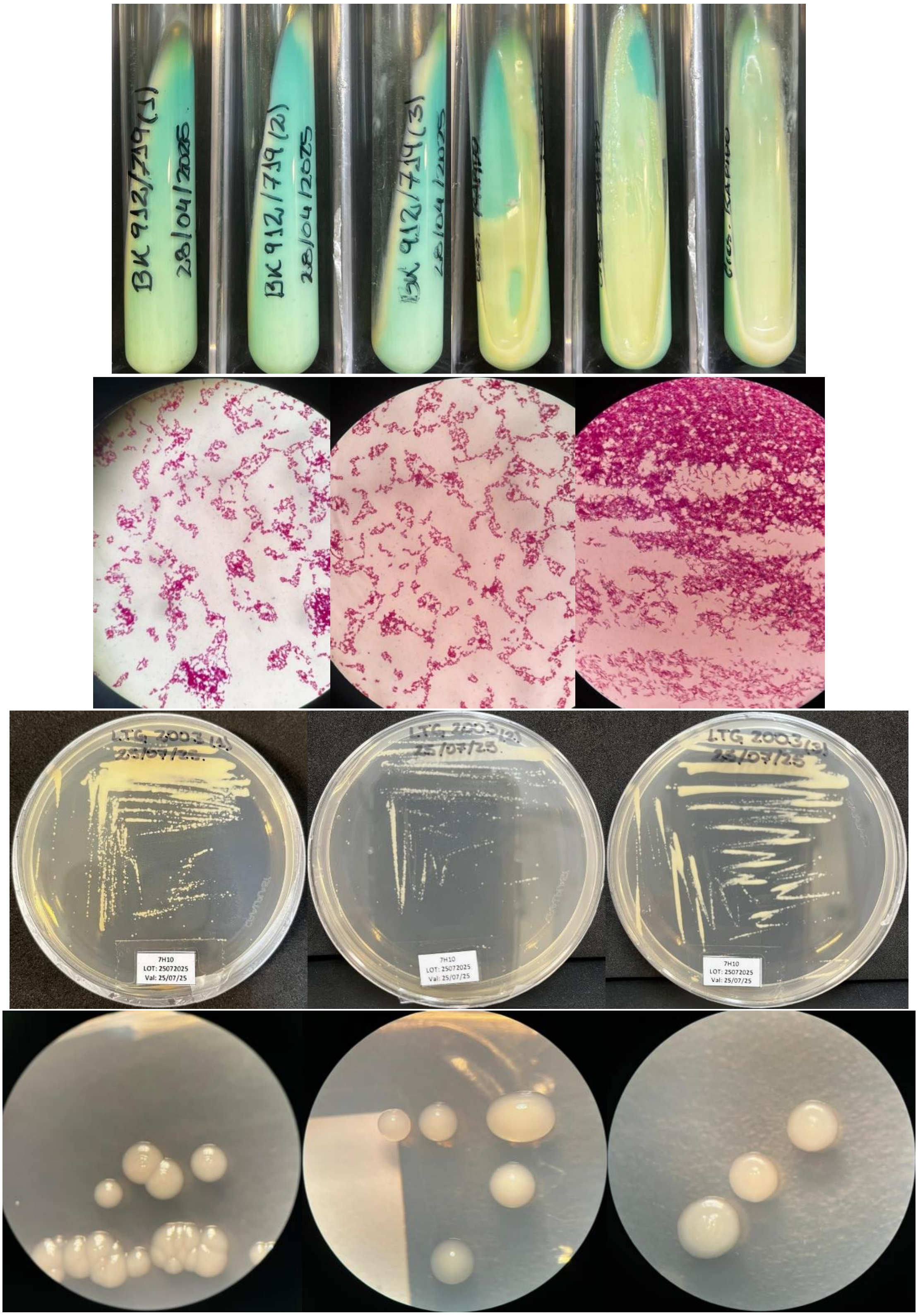

Supplementary figure 1. Morphological images of colonies of isolates LTG2003 (1, 2, and 3), renamed BK912/719 (1, 2, and 3), grown on Löwenthenssen agar at 37 °C (first row). Smears of each strain grown on Löwensen agar at 37 °C using Ziehl-Neelsen (ZN) staining (second row). Images of the strains grown on 7H10 agar (third row). Colonies of each culture on 7H10, captured with a stereomicroscope magnification of 1.6X (fourth row).

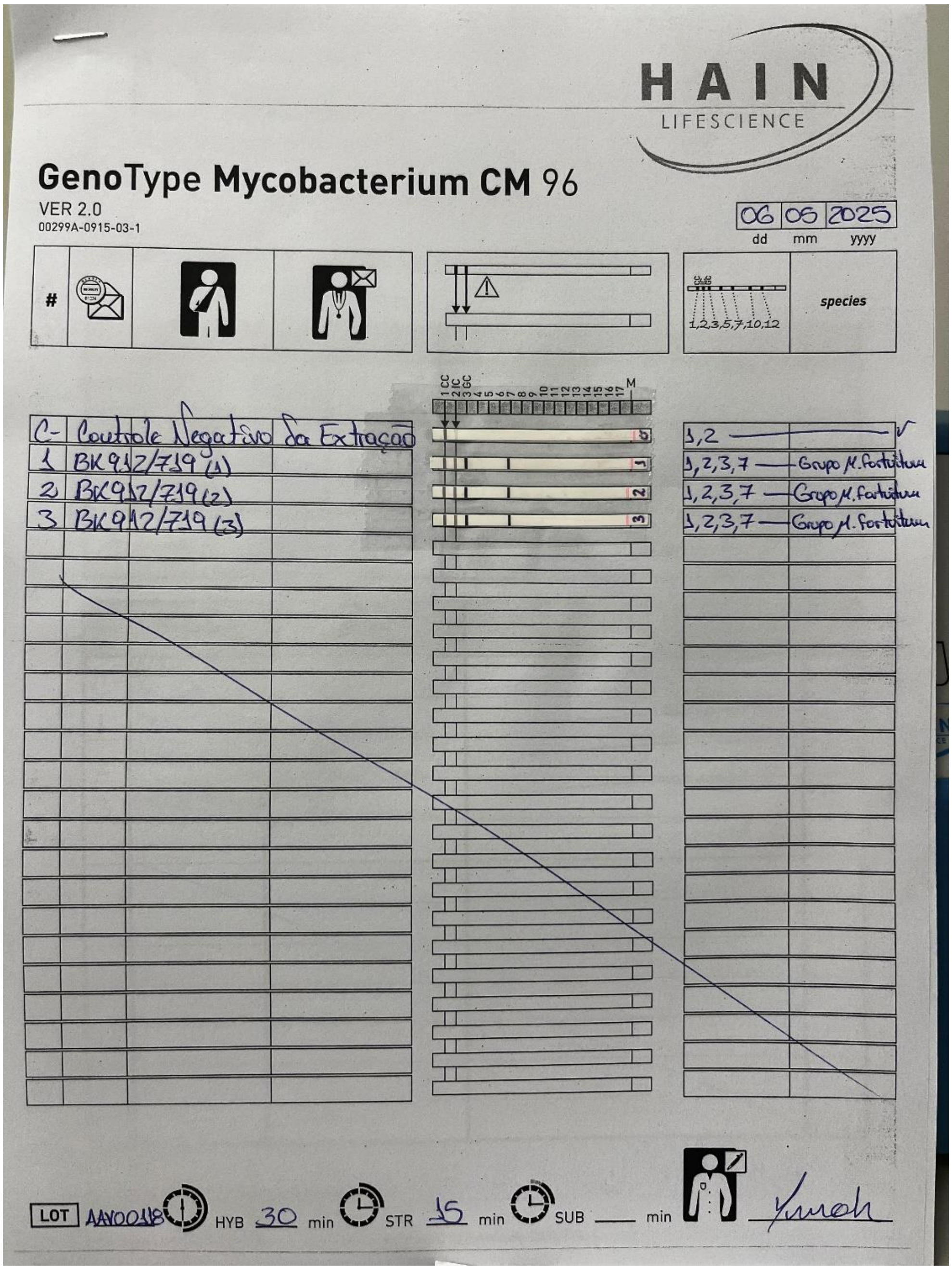

Supplementary figure 2. Results of the Genotype Mycobacterium CM assay (Hain Lifescience) for the LTG2003 culture samples (1, 2, and 3).

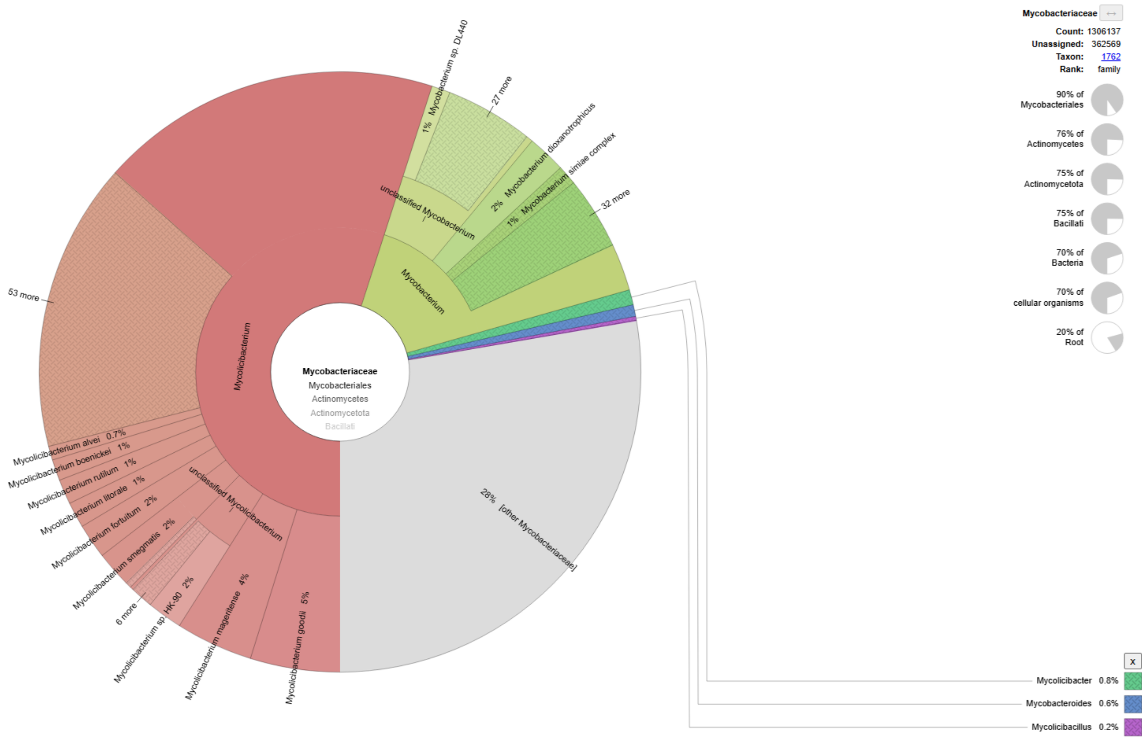

Supplementary figure 3. Results of taxonomic evaluation of the LTG2003$_{(1)}$ genome using the Kraken v2.0 program and Krona.

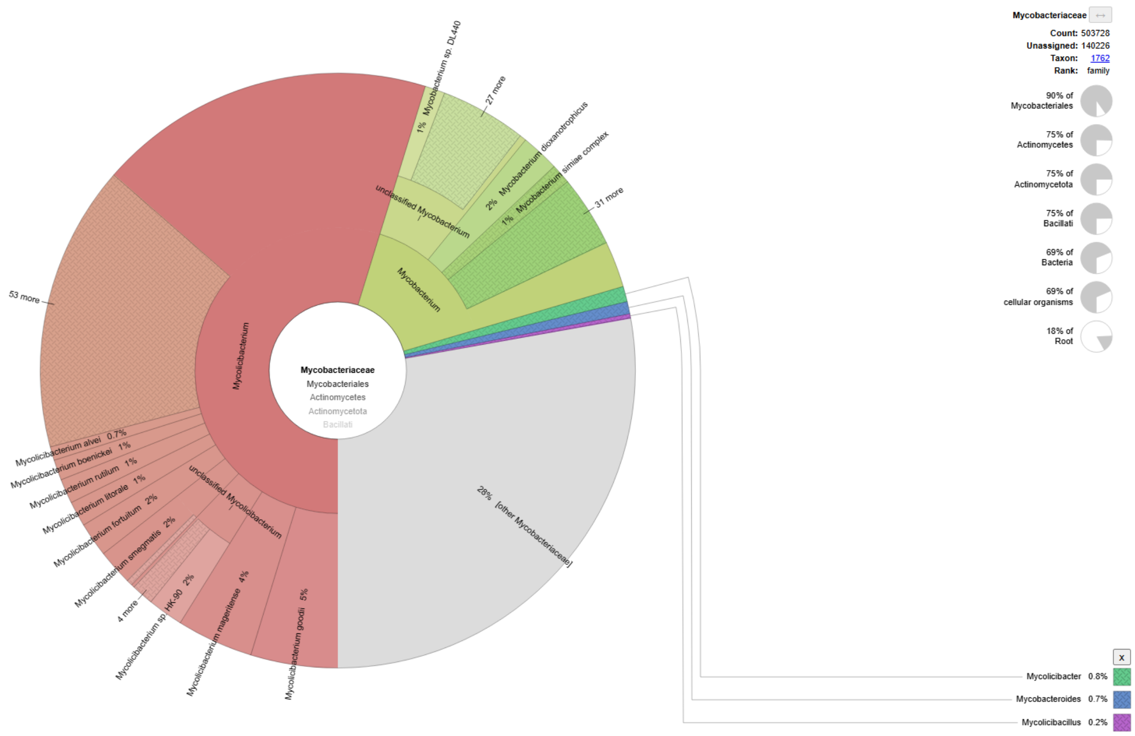

Supplementary figure 4. Results of taxonomic evaluation of the LTG2003$_{(2)}$ genome using the Kraken v2.0 program and Krona.

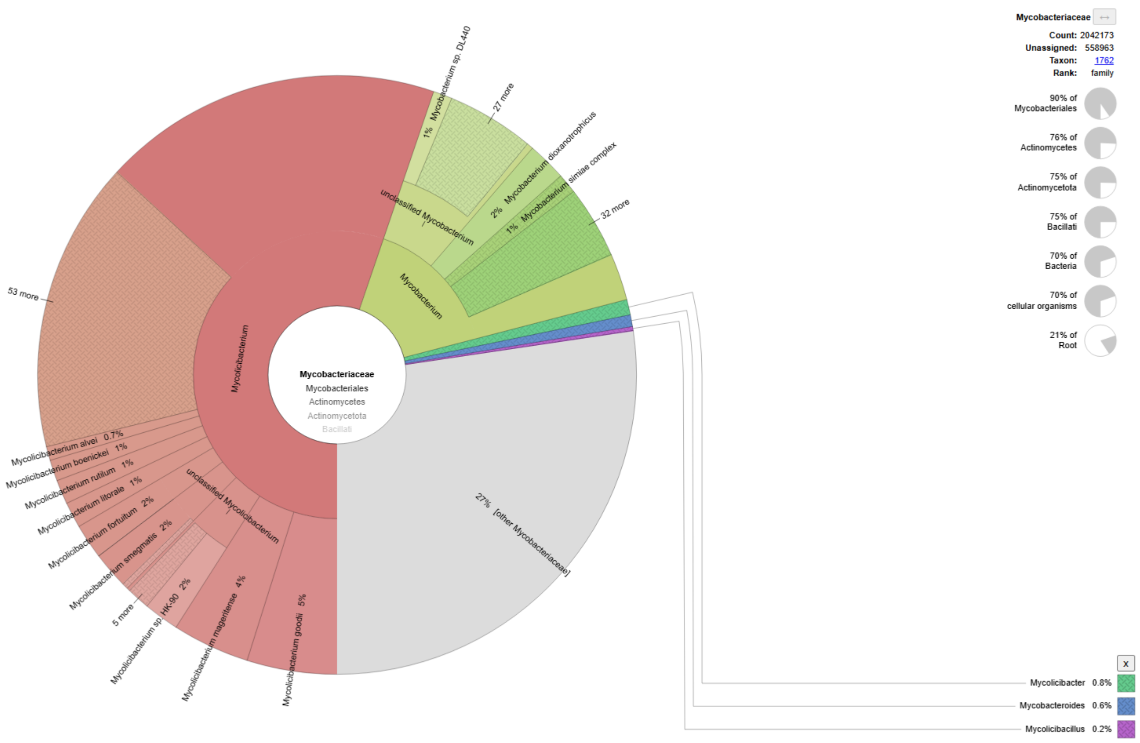

Supplementary figure 5. Results of taxonomic evaluation of the LTG2003$_{(3)}$ genome using the Kraken v2.0 program and Krona.

Supplementary table 1. Characteristics of the antibiotic and toxic compound resistance and the virulence subsystems, comprising of 24 identified genes.

Category	Subcategory	Subsystem	Role	Features
Virulence, Disease and Defense	Resistance to antibiotics and toxic compounds	Beta-lactamase	Metal-dependent hydrolases of the beta-lactamase superfamily III	fig|59750.14.peg.4337
Virulence, Disease and Defense	Resistance to antibiotics and toxic compounds	Mercuric reductase	PF00070 family, FAD-dependent NAD(P)-disulphide oxidoreductase	fig|59750.14.peg.1114
Virulence, Disease and Defense	Resistance to antibiotics and toxic compounds	Bile hydrolysis	Choloylglycine hydrolase (EC 3.5.1.24)	fig|59750.14.peg.3457
			Bile acid 7-alpha dehydratase BaiE (EC 4.2.1.106)	fig|59750.14.peg.514
Virulence, Disease and Defense	Resistance to antibiotics and toxic compounds	Copper homeostasis	Copper-translocating P-type ATPase (EC 3.6.3.4)	fig|59750.14.peg.863, fig|59750.14.peg.3919, fig|59750.14.peg.3987, fig|59750.14.peg.3993, fig|59750.14.peg.3996, fig|59750.14.peg.6693
Virulence, Disease and Defense	Resistance to antibiotics and toxic compounds	Copper homeostasis	Multidrug resistance transporter, Bcr/CflA family	fig|59750.14.peg.6735
			Copper resistance protein CopC	fig|59750.14.peg.883
			Copper resistance protein CopD	fig|59750.14.peg.882, fig|59750.14.peg.2080

Virulence, Disease and Defense	Resistance to antibiotics and toxic compounds	Cobalt-zinc-cadmium resistance	Transcriptional regulator, MerR family	fig|59750.14.peg.22, fig|59750.14.peg.1425, fig|59750.14.peg.2182, fig|59750.14.peg.2381, fig|59750.14.peg.4847, fig|59750.14.peg.5331, fig|59750.14.peg.5542, fig|59750.14.peg.6203
Virulence, Disease and Defense	Resistance to antibiotics and toxic compounds	Resistance to fluoroquinolones	DNA gyrase subunit B (EC 5.99.1.3)	fig|59750.14.peg.4942
Virulence, Disease and Defense	Invasion and intracellular resistance	Mycobacterium virulence operon involved in DNA transcription	DNA-directed RNA polymerase beta' subunit (EC 2.7.7.6)	fig|59750.14.peg.3296
			DNA-directed RNA polymerase beta subunit (EC 2.7.7.6)	fig|59750.14.peg.3297
Virulence, Disease and Defense	Invasion and intracellular resistance	Mycobacterium virulence operon involved in an unknown function with Superoxide dismutase	FIG037441: Conserved transmembrane protein	fig|59750.14.peg.891
			FIG017534: hypothetical protein	fig|59750.14.peg.892
			Superoxide dismutase [Mn/Fe] (EC 1.15.1.1)	fig|59750.14.peg.893
Virulence, Disease and Defense	Invasion and intracellular resistance	Mycobacterium virulence operon involved in protein synthesis (LSU ribosomal proteins)	LSU ribosomal protein L20p	fig|59750.14.peg.993
Virulence, Disease and Defense	Invasion and intracellular resistance	Mycobacterium virulence operon involved in protein synthesis (LSU ribosomal proteins)	Translation initiation factor 3	fig|59750.14.peg.995
			LSU ribosomal protein L35p	fig|59750.14.peg.994
Virulence, Disease and Defense	Invasion and intracellular resistance	Mycobacterium virulence operon possibly involved in quinolinate biosynthesis	L-aspartate oxidase (EC 1.4.3.16)	fig|59750.14.peg.5354
			Quinolinate phosphoribosyltransferase [decarboxylating] (EC 2.4.2.19)	fig|59750.14.peg.5355
Virulence, Disease and Defense	Invasion and intracellular resistance	Mycobacterium virulence operon possibly involved in quinolinate biosynthesis	Quinolinate synthetase (EC 2.5.1.72)	fig|59750.14.peg.5353
Virulence, Disease and Defense	Invasion and intracellular resistance	Mycobacterium virulence operon involved in protein synthesis (SSU ribosomal proteins)	Translation elongation factor G	fig|59750.14.peg.5115
			SSU ribosomal protein S12p (S23e)	fig|59750.14.peg.5117
			FIG025093: Probable membrane protein	fig|59750.14.peg.3556

			Translation elongation factor Tu	fig|59750.14.peg.5114
Virulence, Disease and Defense	Invasion and intracellular resistance	Mycobacterium virulence operon involved in DNA transcription	DNA-directed RNA polymerase beta subunit (EC 2.7.7.6)	fig|59750.14.peg.3297
Virulence, Disease and Defense	Invasion and intracellular resistance	Mycobacterium virulence operon involved in an unknown function with Superoxide dismutase	FIG037441: Conserved transmembrane protein	fig|59750.14.peg.891
			FIG017534: hypothetical protein	fig|59750.14.peg.892
			Superoxide dismutase [Mn/Fe] (EC 1.15.1.1)	fig|59750.14.peg.893
Virulence, Disease and Defense	Invasion and intracellular resistance	Mycobacterium virulence operon involved in protein synthesis (LSU ribosomal proteins)	LSU ribosomal protein L20p	fig|59750.14.peg.993
			Translation initiation factor 3	fig|59750.14.peg.995
			LSU ribosomal protein L35p	fig|59750.14.peg.994
Virulence, Disease and Defense	Invasion and intracellular resistance	Mycobacterium virulence operon possibly involved in quinolinate biosynthesis	L-aspartate oxidase (EC 1.4.3.16)	fig|59750.14.peg.5354
			Quinolinate phosphoribosyltransferase [decarboxylating] (EC 2.4.2.19)	fig|59750.14.peg.5355
			Quinolinate synthetase (EC 2.5.1.72)	fig|59750.14.peg.5353
Virulence, Disease and Defense	Invasion and intracellular resistance	Mycobacterium virulence operon involved in protein synthesis (SSU ribosomal proteins)	Translation elongation factor G	fig|59750.14.peg.5115
			SSU ribosomal protein S12p (S23e)	fig|59750.14.peg.5117
			FIG025093: Probable membrane protein	fig|59750.14.peg.3556
			Translation elongation factor Tu	fig|59750.14.peg.5114
			SSU ribosomal protein S7p (S5e)	fig|59750.14.peg.5116
